# ETAM: Next Generation Event Timer for Picosecond-Precision Time and Amplitude Measurements

**DOI:** 10.3390/s23146380

**Published:** 2023-07-13

**Authors:** Aleksandrs Kalinovskis, Vsevolods Stepanovs, Armands Ancans, Atis Elsts

**Affiliations:** Institute of Electronics and Computer Science (EDI), Dzerbenes 14, LV-1006 Riga, Latvia; aleksandrs.kalinovskis@edi.lv (A.K.); vsevolods.stepanovs@edi.lv (V.S.); armands.ancans@edi.lv (A.A.)

**Keywords:** event timers, precise timing, amplitude measurements

## Abstract

Riga Event Timers have the ability to measure the interval between events with high resolution, on the order of picoseconds. However, they have several drawbacks, such as sensitivity to environmental temperature changes and an inability to capture the amplitude of the events. In this work, we present the ETAM: a next generation Event Timer. Its innovative features include adaptive correction of measurement errors based on an internal temperature sensor, and integrated peak-detector circuit to determine the amplitude of nanosecond-duration pulses. Evaluation shows that the ETAM has high thermal stability with a root mean square error (RMSE) of <3 ps in a temperature range between 0 and +40 °C, and accurate event amplitude measurement capability, with <2.3 mV RMSE in the 100–1000 mV range. These improvements allow the ETAM to be used in satellite laser ranging, optical time-domain reflectometry, and other field applications that require temperature- and amplitude-based time correction in addition to high robustness, performance, and stability.

## 1. Introduction

Event Timers (ET) are high-precision electronic devices that measure the time interval between two events (i.e., voltage pulses) with extreme precision. Existing ET such as A033-ET and A040-ET are characterized by 3 to 4 ps root-mean square error (RMSE) for single-shot pulses [1], in stable operating conditions. These timers are used in scientific applications such as satellite laser ranging (SLR) [2,3,4,5] and optical time domain reflectometry (OTDR) [6,7]. For example, in SLR, the Event Timers are used to measure the time delay between the emission of the laser pulse and its reception at the ground station. Picosecond-precision time resolution is required for SLR applications, e.g., the study of Earth’s gravity field.

However, the existing Event Timers have certain limitations. The measurement precision is negatively affected by changes in the ambient temperature. This requires stable operating environments, which are difficult and expensive to achieve. In addition, the events that need to be captured by the timer in SLR and other applications have high dynamic ranges. The precise time an event exceeds the ET’s sensitivity threshold and is registered by the ET therefore depends on the event’s amplitude, rather than just the timing of its peak. This is called the time-walk effect; an amplitude-based compensation mechanism is typically used to remove it from the measurement results. However, using an amplitude-measurement device complicates the setup and leads to a lengthy dead time after each event. When SLR measurements are taken during the daytime, noise spikes caused by ambient daylight register as frequent low-amplitude events, and therefore the dead time should be minimized.

**Figure 1:**
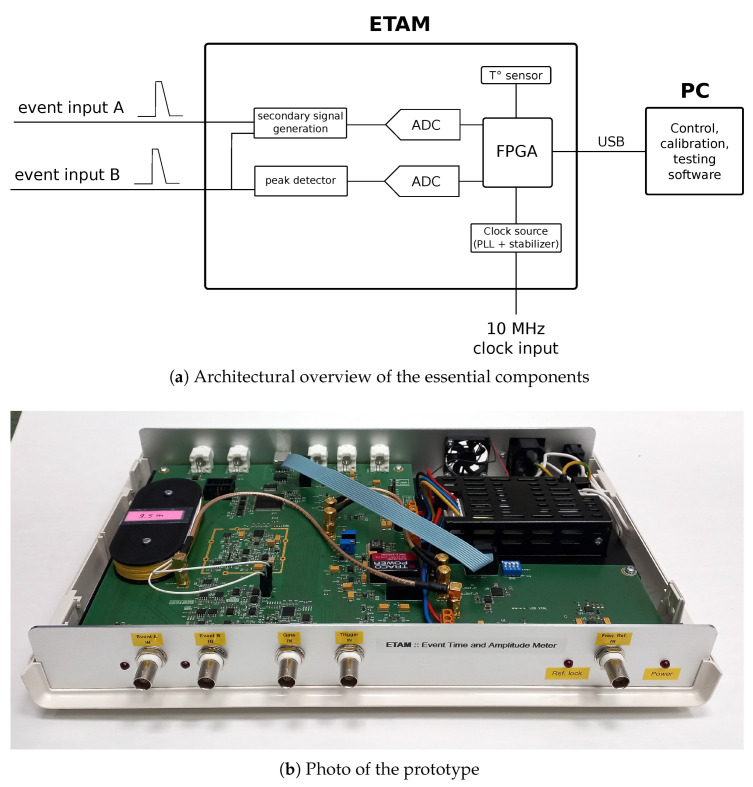
Architectural overview (simplified) and photo of the ETAM.

We present the ETAM (Event Time and Amplitude Meter) (Figure 1): a next-generation ET with built-in amplitude measurement functionality. It features two novel contributions:

1. Adaptive compensation of the measurement error based on temperature readings, with the goal to increase measurement stability under ambient temperature changes.
2. Integration of a peak-detector-based circuit for accurately determining the amplitude of nanosecond-duration pulses with low dead time.

Previous ET models require recalibration whenever their operating temperature changes. The ETAM includes a temperature sensor and uses a dynamic compensation method parameterized by the timer’s internal temperature. The method uses calibration tables built during the manufacturing process, which cycles the ETAM through a range of temperatures in a controlled manner. This method ensures high stability under changing ambient conditions.

The ETAM (Figure 1) supports two analog inputs with user-configurable event-detection thresholds and polarities (i.e., detecting either rising or falling edge), in contrast with the previous ET which supported only digital inputs. In addition, a high-stability 10 MHz clock input is required for accurate measurements, although the ETAM also includes an internal crystal oscillator and can be operated without this external clock. The signal processing in the ETAM is based on a high-accuracy analog-to-digital converter (ADC) and a powerful Field Programmable Gate Array (FPGA). Finally, the ETAM is connected to a controller PC through a standard USB 2.0 interface, providing a convenient and flexible means of data transfer and measurement control.

Experiments show that the ETAM achieves a 2.1–2.4 ps RMSD in a stable environment. We evaluated the ETAM’s architecture in a temperature chamber and found that its timing precision stability is high, with a <3 ps RMSD in the 5–40 °C range. In terms of amplitude measurement, ETAM decreases the dead time to around 40 ns, and achieves an accuracy of 2.3 mV RMSD for pulses with duration between 700 ps to 100 ns. These results are all improvements over previous-generation ET and will enable higher accuracy SLR measurements and other applications.

The paper is structured as follows: Section 2 presents a background on Event Timers. Section 3 presents the evolution of the ETAM, its design, operation and performance summary. Section 4 describes the measurement stabilization method that allows accurate results to be achieved in a wide range of operating temperatures. Section 5 covers the amplitude measurement technique used in the ETAM. Finally, Section 6 concludes the paper.

## 2. Background and Related Work

### 2.1. Enhanced Event Timing

The principles of the Enhanced Event Timing (EET) were presented by the late Dr. Artyukh in [8]. Rather than trying to measure an event (in essence, amplitude spikes) on the input directly, EET relies on the generation of a secondary signal with specific properties. By measuring the parameters of the secondary signal, the time of the event can be measured very precisely even without an ultra-fast ADC. The initial work reported an error of 10–20 ps [8] for single-shot measurements; this error was reduced to <5 ps in subsequent models of Event Timers [1,9].

**Figure 2:**
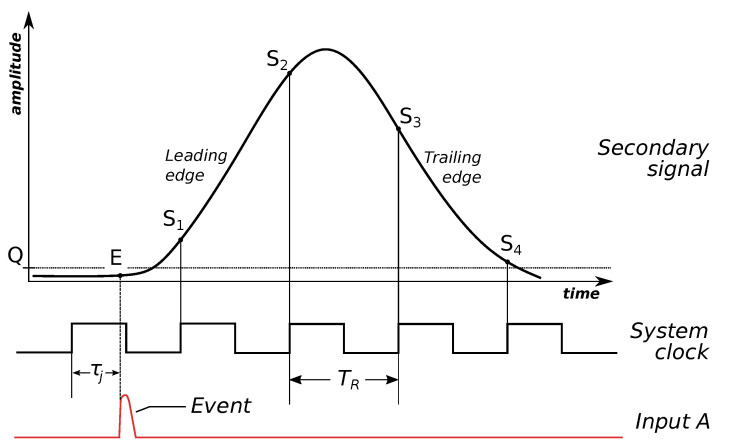
The principles of the Enhanced Event Timing method [8]. The figure shows the shape of the secondary signal, in relation to the timing of the input pulse (event) and the system clock.

Figure 2 shows a graphical overview of the EET method. The input *A* is connected to a secondary signal generator. Once the amplitude on the input exceeds some fixed threshold, a response signal with a predefined shape is generated. In this way, the secondary signal is asynchronous to the system’s clock, but in phase with the event’s signal. The secondary signal amplitude is continuously monitored by an on-board circuitry. Once the amplitude exceeds its own threshold *Q*, a measurement sequence is started using the system’s ADC, and signal amplitudes at points $S_{1}$, $S_{2}$, $S_{3}$, $S_{4}$ are obtained. The secondary signal has a fixed shape; therefore, by measuring the amplitude in any of the points, the phase of the signal can be recovered. This phase corresponds to the fine-grained time resolution of the event. To remove ambiguity about the timing of the event, the system clock cycle counter is used as the coarse-grained part of the measurement timestamp. Together they constitute a precise and unambiguous record of the event’s timing.

More formally, the timestamp of the *j*-th input event is measured as [8]:

(1) $$t_{j}=N_{j}T_{R}+\tau \left(S_{j1}\right),$$

where $T_{R}$ is the period of the system’s clock pulse (10 ns for a typical 100 MHz system clock), $N_{j}$ is the number of the system’s clock pulses at the time of the event, $S_{j1}$ is the digitized amplitude measurement of the secondary signal generated after the event (with amplitude threshold greater than or equal to *Q*), and τ(u) is a calibration function. The calibration function is obtained in a specific calibration process; the process uses the assumption that the shape of the secondary signal is fixed, therefore its measured amplitude only depends on the phase of the secondary signal, and therefore on the timing of the event itself. We also note that in the ETAM device, only the amplitude measured in the point $S_{1}$ is used as the argument of the calibration function τ(u). Previous Event Timers used measurements on both the leading edge and trailing edge of the secondary signal, in up to four points $S_{i}$. While this is still possible with ETAM, this functionality is not required to achieve the accuracy we report in this paper.

The EET technology is used in the previous-generation Event Timers, such as A033-ET and A040-ET [1,9], which are installed in a large number of the world’s SLR stations at the time of writing this manuscript (https://eventechsite.com/en/applications/, accessed on 7 July 2023). The accuracy of our existing Event Timers is comparable with the best industry analogues, such as GuideTech’s GT668PCI-1 (https://www.guidetech.com/gt668pci-1/, accessed on 7 July 2023) (2 ps single-shot resolution) and research prototypes such as the work by Panek et al. [10]. However, both the existing event timers and the existing comparable devices do not have good temperature stability or the capability to measure amplitude of single nanosecond-duration pulses.

### 2.2. Timing Measurement Stability under Changing Ambient Conditions

It is well known that the accuracy of timing-related hardware such as clock sources and measurement circuits are susceptible to changes in ambient conditions. According to IEEE standard P1193 [11], the most important effects are “acceleration (including vibration), temperature, humidity, barometric pressure, load impedance, power-supply voltage, electric and magnetic fields, and radiation”. Other factors such as particle pollution may impact these aspects, and thus indirectly contribute to timing errors.

From the IEEE list, *temperature* is the key environmental impact of interest for our applications. In SLR and optical cable monitoring, vibrations can be largely avoided, and the effect from the other factors except T° is much smaller or can be controlled locally, for example, by hermetically sealing the case of the Event Timer.

There are multiple approaches to reduce the impact from ambient temperature dynamics. According to Artyukh et al. [9] these are:

- temperature stabilization at the point of the measurement;
- temperature effects compensation in software;
- improved calibration procedure.

While specific electronic components can be temperature-stabilized quite easily—for example, oven-controlled oscillator crystals (OCXO) can be readily purchased—the problem is that the temperature has an impact on every component in the signal and secondary signal pathways. Placing the entire Event Timer in a temperature-controlled casing is possible, but cumbersome, and Dr. Artyukh reports [9] that “neither thermostat with heating elements nor thermostat with cooling on the base of Peltier elements could provide a sufficient precision stability, and were not the best solution in terms of hardware simplicity, compactness and power consumption”.

The approach selected in this work is therefore a compensation of temperature effects through improved calibration; this approach is described and evaluated in detail in Section 4. It is similar to those used in, e.g., [12,13]. However, the focus of these previous works is to compensate for inaccuracies of the crystal oscillator itself, with the goal to apply the devices in a standalone mode in, e.g., satellite on-board applications. Our work, in contrast, assumes that a high-quality external clock input is provided (e.g., a signal from a GPS-disciplined oscillator), and the main goal is to compensate for the temperature impact on the secondary signal parameters.

### 2.3. Amplitude Measurements

Peak power detection of nanoseconds-duration pulses is required in many application areas, including medicine [14], spectroscopy [15], telecommunications, nuclear technology, security and ultra-precise measurements of optical pulses [7]. In the event timer context they have multiple applications:

- Estimate the power of received signals, giving important information about the event.
- Filter out spurious events that have been registered due to noisy input signals.
- Apply amplitude-based correction to event timestamps.

**Figure 3:**
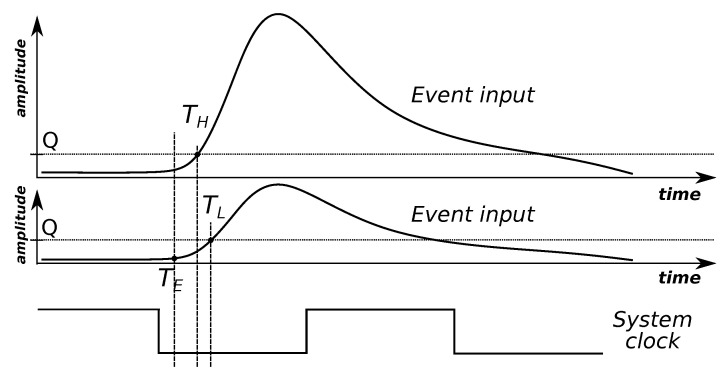
Due to a fixed sensitivity threshold *Q*, the timestamp of an event depends on its amplitude. An event that occurs at the time $T_{E}$ is recorded at an earlier time $T_{H}$ if it has higher amplitude, but at a later time $T_{L}$ if it has lower amplitude.

To explain the last application in more detail: higher-amplitude events are likely to register as earlier compared with lower-amplitude events (Figure 3). This is because the secondary signal is generated at a user-configurable, but fixed input signal threshold level. The so-called time-walk effect results in event timing being depedent on its amplitude.

Several options exist that allow compensation for this time-walk effect. One method is to use a constant fraction discriminator (CFD); however, they have time-walk effect on their own [16]. Another method is to measure the amplitude of the event, and apply a correction coefficient proportional to the amplitude. The amplitude of nanosecond-duration pulses cannot be measured easily with an ADC, as an extremely high (above GHz) sampling frequency would be required. Therefore, alternative methods such as single-pulse peak detector (SPD) are typically used. The principle of single-pulse peak detector are well understood; see for instance the original work [17] and a recent review on the topic [18].

For instance, the Riga Satellite Laser Ranging station currently uses a Time Selector/Amplitude-to-Time Interval Converter (TS/ATIC) system [19]. The system splits the input signals in two parts; it then feeds one part to an Event Timer, and the other to a peak-detector based device for measuring the amplitude. However, ATIC’s method of amplitude measurement is hindered by a lengthy (microsecond) dead-time, which is a problem especially in the daytime, when there are many spurious events due to atmospheric noise. The detector used in the Riga SLR has a narrow gate width of ≈2 μs, during which the TS/ATIC system can only measure two events, while the ETAM will be able to measure 10–15 events.

Our own previous work on this topic (Aristov et al. [20]) presents a detailed analysis of SPD-based amplitude measurement. However, the current paper presents a peak detector fully integrated in the Event Timer, along with experimental results from such a device.

## 3. ETAM: Next Generation Event Timer

### 3.1. Design of the ETAM

Figure 1a shows a high-level non-comprehensive schematic overview of the ETAM. It has two event inputs *A* and *B* and one 10 MHz reference clock input (some non-essential inputs and outputs are not shown in the image). The external clock is expected to be extremely stable in order to achieve the specified timing precision. In the absence of an external clock, the internal crystal oscillator can also be used; however, high precision is then not guaranteed. The 10 MHz clock input, if present, is up-converted in the internal Phase Locked Loop (PLL) to a 100 MHz clock source, which is supplied to the FPGA and other digital logic in the device. The stabilized PLL is implemented on a daughter-board connected with the main board of the ETAM via extension headers for control interface and coaxial cables for clocks.

The main board of the ETAM features all the components required digital and analog signal processing logic. There is a secondary signal generation circuit; following the EET principles, it is used to create a fixed-parameter signal. The event input *B* is also connected to a peak detector circuit (Section 5). ETAM measures the amplitude of events by digitizing the peak-detected signal. This built-in amplitude measurement functionality allows to correct the timing interval measurement results in a post-processing step, as well as to record additional information about the event itself. Two high-speed ADC circuits (12 and 14 bit) are present for sampling the secondary signal and the peak-detectors output, respectively. The ADC are controlled by a FPGA chip (Intel/Altera Cyclone III), which in turn is driven by the system clock coming from the PLL daughter-board. Finally, the main board features a communication interface (USB 2.0) between the FPGA and the PC, which controls and receives measurements from the ETAM.

A noteworthy feature is the on-board temperature sensor, implemented via an off-the-shelf integrated circuit. Its design requirements are rather low, approx. ±0.25 °C precision and ±1 °C accuracy, due to the fact that the temperature sensor is used to compensate for event timing precision and accuracy drift, which by themselves are rather stable in temperature and would not benefit from higher accuracy temperature measurements. The secondary purpose of the on-board temperature sensor is to monitor device temperature during testing, where a higher accuracy external sensor can be used if necessary.

### 3.2. ETAM Operation

The following is a brief summary of event processing with the ETAM. The starting point is when an event arrives on one of the inputs *A* or *B*. When the input amplitude exceeds a preconfigured threshold, the secondary signal generation circuitry kicks in and generates a triangle-shaped analog signal on its output. If the event has arrived on the input *B*, the peak-detector circuit is enabled and starts its sample-and-hold operation. It outputs a signal corresponding to the maximum of voltage on input *B*.

The ETAM is controlled by an FPGA chip, which also samples the ADC. Sampling of the secondary signal is triggered by the signal level reaching a preconfigured threshold, while ADC sampling of the peak detector output is triggered by trailing edge of the pulse input signal. After sampling of both has been completed, the FPGA resets the the peak detector (the timing circuit is self-resetting).

Once the event is detected, the next step is to compute the timestamp of the event. The timestamp consists of two parts (Section 2.1). The coarse-grained part is the number of system clock ticks since the star of the timer. The fine-grained part is the offset of the event with respect to the system clock tick where it took place. This part is obtained via a lookup in a calibration table. The table maps each possible above-threshold value from the ADC to a timing offset. The details of the calibration process (i.e., how the table is constructed) are described elsewhere, for instance [8] presents one method to calibrate an Event Timer.

The FPGA also continuously monitors the internal temperature, and when a significant deviation is detected, the host software replaces the current calibration table with the one that matches the current temperature (Section 4).

Finally, the PC to which ETAM is connected receives the stream of timestamps, and using its control and measurement software, applies another set of corrections (e.g., based on the amplitudes of the events). It then expresses the time stamp in a relevant time scale, such as UTC or PC system time.

### 3.3. ETAM Performance

Table 1 shows the expected performance of the ETAM, based on preliminary test results. The results demonstrate improvements both in timing precision and in temperature stability. Moreover, the ETAM features reduced dead-time for the timing measurement itself, not to mention the additional amplitude measurement functionality.

## 4. Measurement Error Stabilization

### 4.1. Overview

The EET method requires a calibration function τ(u) that converts ADC readings to fine-grained timestamps. This function is implemented as a lookup table, using the fact that only a finite number of ADC readings are possible. At the core, the construction of a single ETAM calibration table happens the same way as for the previous-generation Event Timers [8]. However, previous-generation ET require a recalibration whenever environmental temperature changes, in order to maintain precise timing, due to the fact that the shape of the secondary signal is affected by environmental conditions.

In contrast, the ETAM production process now includes the creation of numerous different calibration tables, one for each potential operating temperature, with one-degree Celsius interval. More formally, during the production process the ETAM’s manufacturer is building a set of $\tau_{T}\left(u\right)$ functions parameterized by a temperature parameter *T*. This parameter *T* takes values from the set $\left\{T_{min},T_{min}+1,\ldots ,T_{max}-1,T_{max}\right\}$°C, where $T_{min}$ is the minimum expected operational temperature and $T_{max}$ is the maximum.

During operation, the ETAM monitors its internal temperature using a built-in temperature sensor (Figure 1a). Whenever the ETAM detects a shift greater than 0.5 °C from the calibration temperature of the current table, it switches to another table, better corresponding to the new operational temperature. If the temperature goes below the expected minimum expected operational temperature, the table for $T_{min}$ is used; similarly, the $T_{max}$ table is used for temperatures above $T_{max}$.

The calibration tables are presently stored in the non-volatile memory of the PC that controls the ETAM. This is due to their relatively large size: tens of kilobytes per table for a 14-bit ADC. We are exploring alternative methods, such as polynomial approximation and other compression techniques, to make it possible to fully store all calibration settings in the on-board FPGA.

### 4.2. Metrics

The main stability metrics of an Event Timer are:

- *precision drift*: changes in random error with changes in ambient temperature;
- *epoch drift*: shift in time scale with changes in ambient temperature;
- *offset drift*: change of relative delays of input channels with changes in ambient temperature.

Previous-generation ET has features for stabilizing each of these parameters to some extent, however field applications with environments significantly harsher than climate-controlled laboratory, such as on-site optical cable monitoring, still pose a serious challenge for measurements with high precision and accuracy requirements.

Precision is currently stabilized by the self-calibration procedure and construction of many calibration tables, as described above. Additionally, the ETAM features a new ADC chip in the time-to-digital conversion circuitry, and the rest of the circuitry has received a major update, leading to a significantly more stable gain.

Epoch drift in the earlier ET models is passively compensated in hardware, by carefully selecting a thermistor for each unit. The new ETAM corrects both for the precision decrease (random error) and epoch drift (systematic error) with changing temperatures using the same method, the multiple table-based calibration approach. However, both the new and the old method does not account for component aging, and so requires periodic re-calibration throughout the lifetime of the device. The improved interpolation circuit discussed earlier is also expected to have a positive, albeit small effect on epoch stability.

Offset drift is mitigated by circuit design—the two ETAM input channels are made as similar as possible and are joined into one signal path as early as possible. This design approach, in combination with use of high-speed integrated circuits, makes propagation delays for each channel, and, consequently, their difference, very small: on the order of a nanosecond. The resulting inter-channel delay (i.e., offset) stability is proportional to the delay itself with values of <200 fs/°C, which is negligible compared with offsets introduced by the external equipment.

### 4.3. Performance Evaluation

**Figure 4:**
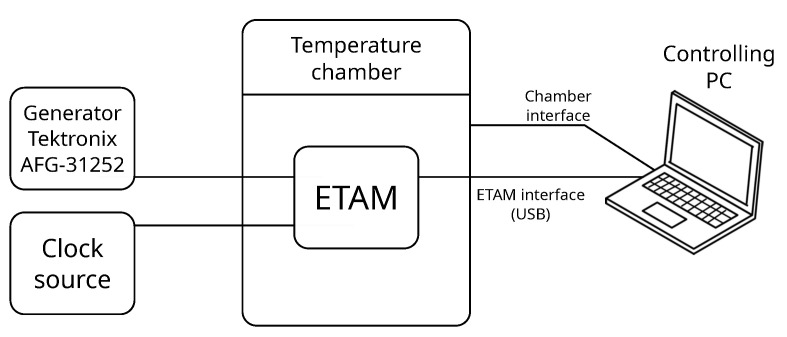
Calibration and testing setup for changing environmental temperature experiments.

During the experiments, the ETAM under test is placed in a thermal chamber and the rest of equipment outside it (Figure 4). Tektronix AFG-31252 arbitrary function generator is used to generate the calibration and testing signals. The signal is generated in two channels, connected to the ETAM’s input channels A and B. The period between pulse events is set to 10 ms, amplitude to 2.5 V, width to 100 ns. Channel B has a 700 ns lead delay. The experiment consists of two stages described below.

#### 4.3.1. Calibration Table Collection

The Event Timer and PC software executes the following algorithm:

1. Set temperature chamber to a predefined temperature, with a 1 °C step.
2. Wait while the T° in the chamber matches the preset value.
3. Run the calibration procedure using the generator’s signal as a reference.
4. Save the calibration table for the specific temperature.

#### 4.3.2. Timing Precision Testing

The Event Timer and PC software executes the following algorithm:

1. Set temperature chamber to a predefined temperature, with a 0.5 °C step.
2. Measure the T° in the thermal chamber and the internal T° of the ETAM.
3. Apply the calibration table corresponding to the internal T° of the device.
4. Accumulate 4000 time measurements in a batch.
5. Save, process and display the measurement results.

**Figure 5:**
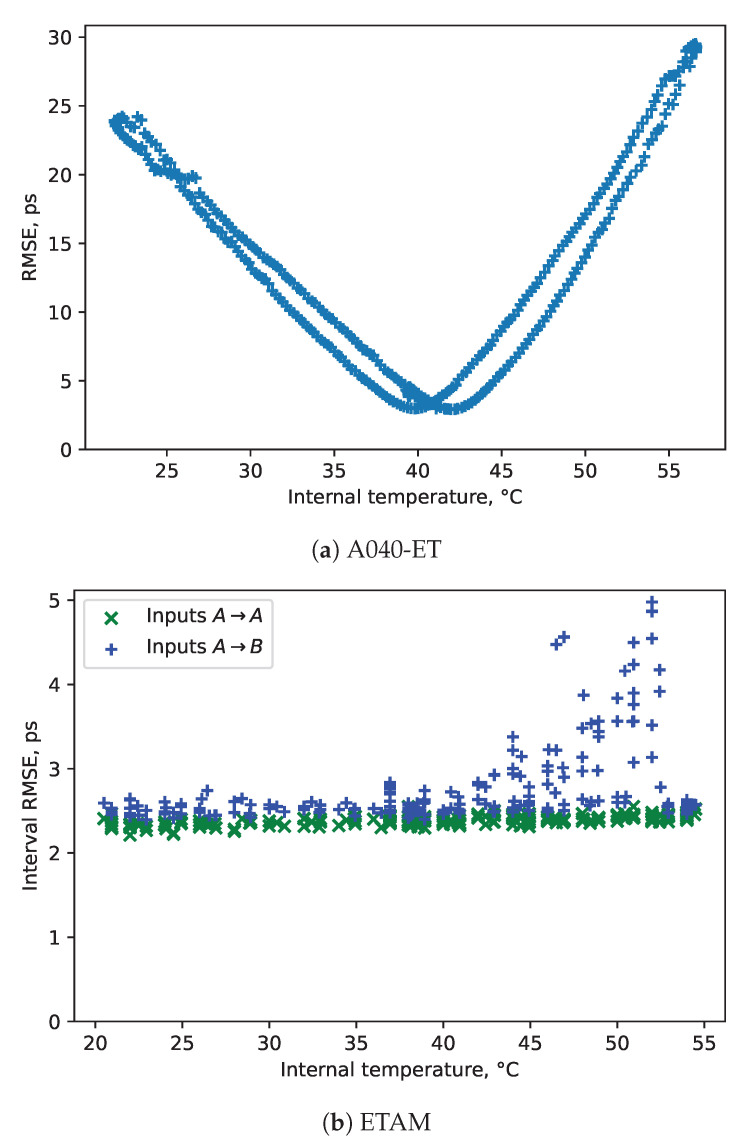
Timing error depending on T°, under a range of temperature settings.

The summary results of the tests are provided in Table 1. Figure 5 shows the dependence on the precision error on the temperature for the ETAM and the previous-generation event timer. The previous-generation timer has a clear dependence between the error and the internal temperature. In contrast, the ETAM has nearly constant error in the intra-channel event timing (input A to A), and small error in the inter-channel event timing (input A to B). These are preliminary results of the first prototype, and it is likely that the increase inter-channel error will be reduced in the future.

## 5. Amplitude Measurements

### 5.1. Motivation

The measurement of amplitude for short pulses poses a significant technical challenge. To accomplish this, the Riga Satellite Laser Ranging (SLR) station currently utilizes a combination of Event Timing technology and an Amplitude-to-Time Interval Converter (ATIC) [19,21]. The ATIC effectively converts the pulse amplitude into a proportional time interval, which can then be measured using an event timer. However, this method has the drawback of a relatively long dead-time, which typically falls within the range of microseconds.

The new pulse amplitude measurement technology implemented in ETAM reduces it to approximately 40 ns, an order of magnitude improvement compared with the ATIC. Instead of the amplitude-to-time interval conversion, the ETAM employs a high-speed peak detector to capture the pulse amplitude at a higher rate. The dead time for event timing itself also is slightly reduced compared with the previous-generation Event Timers (Table 1). Moreover, the ETAM simplifies the application of Event Timers in the SLR domain and has the potential to reduce costs, due to this integration of amplitude measurements and timing in a single device.

### 5.2. Overview

Direct conversion of pulses with a duration of $\tau_{pulse}$< 2 ns is currently impossible in a cost-efficient way, because this requires an ADC with a sampling frequency on the order of a GHz. If a peak-detector is used instead, it stores the maximum amplitude of the input pulse and holds it for a time sufficient for its digitization with a much slower ADC.

**Figure 6:**
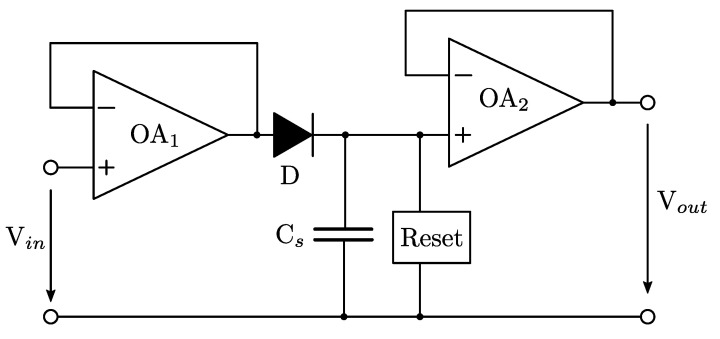
Simplified circuit of the peak detector used in the ETAM.

Figure 6 shows a simplified circuit of the peak detector. In essence, it is of conventional topology first described in [17] and, more recently, in good detail, in [18]. In our work it has been updated with modern high-speed operational amplifiers. The ETAM’s peak detector circuit is composed of two buffer stages. The first one (OA${}_{1}$) is characterized by its high-speed operation, and low output resistance (output capability to quickly charge the storage capacitor C${}_{s}$). The second one (OA${}_{2}$) is characterized by its high input resistance, low input offset voltage and offset current. The circuit also includes a storage capacitor (C${}_{s}$) that exhibits minimal charge leakage, a Schottky diode (D) with high-frequency capabilities of up to 12, GHz and small parasitic parameters, as well as a reset circuitry.

The circuit functions by tracking the input signal voltage (V${}_{in}$) and maintaining the peak value of the signal on the output (V${}_{out}$) until it is reset by the reset circuitry. This feature enables the use of a slower ADC for capturing and digitally processing the output signal. However, this circuit has certain limitations. The bandwidth limitations of the circuit components significantly affect pulses with widths less than 10 ns. Additionally, the voltage drop across D restricts the minimum pulse amplitude that can be captured. Furthermore, the nonlinearity of D introduces substantial pulse width dependency, thereby impacting precise amplitude measurements.

### 5.3. Performance Evaluation

In order to evaluate the performance of the peak-detector for pulse amplitude measurement, we use an experimental amplitude measurement device (AMD) based the peak detector circuit (shown in Figure 6) with a high-precision pulse generator (Tabor Electronics WS835), which can generate arbitrary nanosecond duration pulses.

A single input pulse (or an input pulse with a low repetition frequency) is fed to the AMD from the generator as a source. To characterize the AMDs amplitude measurement performance, an iterative approach is used, where a parameter of the pulse is iteratively changed in a predefined range of values.

#### 5.3.1. Dependence on Pulse Amplitude

The experiment consists of feeding the ETAM pulse signals with fixed parameters and variable amplitude in the operational range from −2 V to −0.05 V (−50 mV).

Since the generated pulse amplitude may deviate from the generator settings, a precise digital multi-meter is used to establish the relationship between generator signal settings and generated voltage. This enables more precise mapping of pulse amplitude settings to their corresponding generated pulse amplitudes $A_{ref}$, accounting for any potential deviations from the generator’s settings. The measurement error here is defined as $\Delta A=A_{m}-A_{ref}$, where $A_{m}$ is the measured amplitude and $A_{ref}$ the reference (“true”) amplitude.

In the experiments, pulses with an amplitude greater than $A_{ref}>-70$ mV were not detected by the AMD. Therefore, it is recommended to limit the range of input pulse amplitudes to between −2 V and −100 mV in order to ensure accurate pulse amplitude registration.

**Figure 7:**
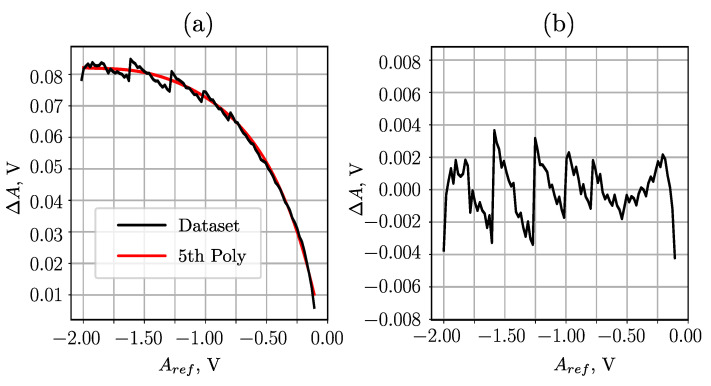
Error ΔA of the ETAM pulse amplitude measurement relative to the reference amplitude $A_{ref}$: (**a**) with raw data, (**b**) with subtracted systematic error.

The measurement error within the defined input pulse amplitude range is depicted in Figure 7. Across the entire range, the difference fluctuates by approximately 80 mV. Nevertheless, a 5th order polynomial fitted to the results provides a reliable approximation of the systematic error. The resulting absolute error is <4.5 mV after compensation.

#### 5.3.2. Dependence on Pulse Width

**Figure 8:**
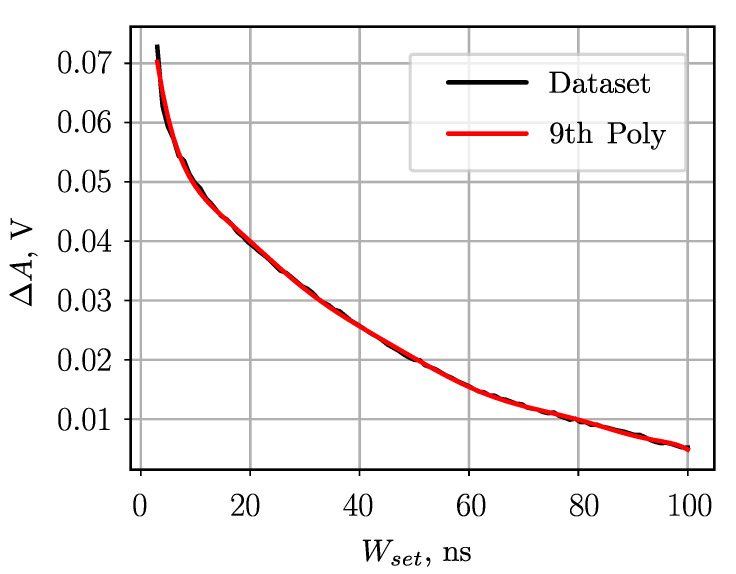
Relation between the variable input pulse **width** and the ETAM amplitude measurement error relative to $A_{ref}=1$ V.

In this experiment, the pulse width setting $W_{set}$ is varied between 1 ns and 100 ns. The amplitude is fixed to 1 V. As shown in Figure 8, in the range 2 ns $\leq W_{set}\leq$ 100 ns the measurement uncertainty is approximately ±35 mV. By using a ninth-order polynomial to compensate for systematic error, the measurement uncertainty could be reduced to approximately ±4 mV. Furthermore, considering the aforementioned approximation and assuming a pulse width precision of ±100 ps, the estimated precision is approximately ±1 mV.

#### 5.3.3. Dependence on Pulse Leading Edge Duration

In this experiment, the goal is to check how the duration of the leading edge of the event impacts the measurement results. To evaluate this, the time of the leading edge $T_{LE}$ for 1 V pulse was changed in the range from 1 ns to 1000 ns (1 μs).

**Figure 9:**
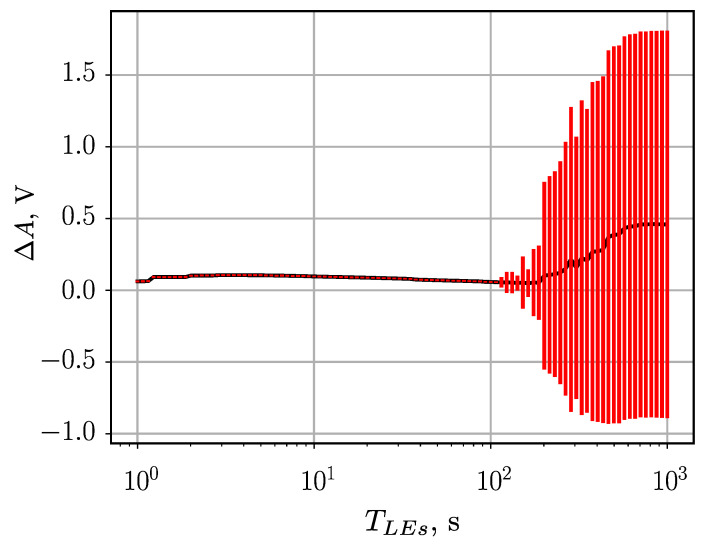
Relation between the variable input pulse **leading edge** and the average error of ETAM pulse amplitude measurements.

For the prototype used in tests, long (>100 ns) leading edge compromises stability of operation of the high-speed comparator integrated circuit, which is used to detect the incoming signal. As shown in Figure 9, this leads to a system malfunction and high apparent measurement error. If application requires working with such slow signals, stability can be easily improved by increasing hysteresis of the comparator circuit. Other than that, the influence of leading edge duration on the measured pulse amplitude is small: ±20 mV for leading edge duration in the 1 to 100 ns range.

#### 5.3.4. Summary

Table 2 shows a summary of the performance. In addition to the experiments presented in detail above, we also study the impact from the trailing edge, period, and delay after enable. To summarize the discoveries and recommendations for the future work:

1. High-accuracy reference for input pulse amplitude is required for calibration of the measurement device.
2. Modification of the peak-detector’s clear-time setting could be used to reduce the lower limit of input pulse period.
3. Extremely slow input pulse leading edges are causing faulty sampling of the peak detector output.
4. The influence of the input pulse leading edge on the measurement results may be related to the pulse signal overshoot in the peak detector.
5. The accuracy and uniformity of pulse amplitude measurements could be improved by processing more ADC samples from the peak detector’s output.

## 6. Conclusions

ETAM is a next-generation Riga Event Timer that features significant evolution on two fronts: increased thermal stability and additional amplitude-measurement functionality. At the moment, three ETAM prototypes have been created. Preliminary evaluation results show <2.6 ps resolution (RMSE) in the operating temperature range 5–40 °C and <4.5 mV amplitude measurement error in the 100 mV to 1000 mV input amplitude range (either positive or negative polarity), with 30 ns dead time. The next steps will include the validation of the ETAM’s performance in real-world SLR and OTDR applications.

## Figures and Tables

**Table 1:** Performance comparison between the Event Timer A040-ET and the new ETAM.

	A040-ET	ETAM
Timing precision (RMSE)	2.5–2.7 ps	2.3–2.5 ps
Timing precision (RMSE) stability	<4 ps (15–30 °C range)	intra-channel: <2.6 ps (5–40 °C) inter-channel: <3 ps (5–25 °C) <5 ps (5–40 °C)
Single-input timing offset drift		<1 ps/°C
Input-to-input timing offset drift		<0.2 ps/°C
Dead time	50 ns	30 ns
Minimum input pulse width	700 ps	700 ps
Amplitude msrmt. range	N/A	±100 mV to ±2 V
Amplitude msrmt. precision (RMSE)	N/A	<4.5 mV (2 V amplitude); <2.3 mV (1 V amplitude)
Amplitude msrmt. accuracy	N/A	<60 mV (any shape and width); <5 mV (tuned for shape and width)

**Table 2:** Amplitude measurement performance summary.

	Range	Error	Error (Compensated)
Input pulse amplitude	−2 V to −100 mV	±60 mV	±4.5 mV
Input pulse width	3 ns to 100 ns	±35 mV	±1 mV
Input pulse leading edge	1 ns to 100 ns	±20 mV	
Input pulse trailing edge	<2 ns	±1 mV	
Input pulse period	10 us to 200 ns	±0.3 mV	
Input pulse delay after enable	>20 ns	±0.3 mV	
